# A Helical Structural Nucleus Is the Primary Elongating Unit of Insulin Amyloid Fibrils 

**DOI:** 10.1371/journal.pbio.0050134

**Published:** 2007-05-01

**Authors:** Bente Vestergaard, Minna Groenning, Manfred Roessle, Jette S Kastrup, Marco van de Weert, James M Flink, Sven Frokjaer, Michael Gajhede, Dmitri I Svergun

**Affiliations:** 1 Department of Medicinal Chemistry, University of Copenhagen, Copenhagen, Denmark; 2 Department of Pharmaceutics and Analytical Chemistry, University of Copenhagen, Copenhagen, Denmark; 3 Biophysics, Novo Nordisk A/S, Bagsvaerd, Denmark; 4 Hamburg Outstation, European Molecular Biology Laboratory, Hamburg, Germany; 5 Institute of Crystallography, Russian Academy of Sciences, Moscow, Russia; California Institute of Technology, United States of America

## Abstract

Although amyloid fibrillation is generally believed to be a nucleation-dependent process, the nuclei are largely structurally uncharacterized. This is in part due to the inherent experimental challenge associated with structural descriptions of individual components in a dynamic multi-component equilibrium. There are indications that oligomeric aggregated precursors of fibrillation, and not mature fibrils, are the main cause of cytotoxicity in amyloid disease. This further emphasizes the importance of characterizing early fibrillation events. Here we present a kinetic x-ray solution scattering study of insulin fibrillation, revealing three major components: insulin monomers, mature fibrils, and an oligomeric species. Low-resolution three-dimensional structures are determined for the fibril repeating unit and for the oligomer, the latter being a helical unit composed of five to six insulin monomers. This helical oligomer is likely to be a structural nucleus, which accumulates above the supercritical concentration used in our experiments. The growth rate of the fibrils is proportional to the amount of the helical oligomer present in solution, suggesting that these oligomers elongate the fibrils. Hence, the structural nucleus and elongating unit in insulin amyloid fibrillation may be the same structural component above supercritical concentrations. A novel elongation pathway of insulin amyloid fibrils is proposed, based on the shape and size of the fibrillation precursor. The distinct helical oligomer described in this study defines a conceptually new basis of structure-based drug design against amyloid diseases.

## Author Summary

Diseases associated with the presence of amyloid structures, such as Alzheimer and Parkinson disease, are characterized by the presence of protein aggregates in the form of highly ordered fibrils. This amyloid fibril formation is also commonly observed for a number of protein drugs, such as insulin. Detailed information on how and why these fibrils are formed will be very useful to design compounds and drugs that may reverse or even prevent fibril formation, but existing knowledge in this field is still limited. We have studied, in real time, the fibril formation of insulin using a technique based on scattering of x-rays (small-angle x-ray scattering [SAXS]). Using SAXS, we obtained hitherto unprecedented three-dimensional structural information on these fibrils in solution. Most importantly, we were able to describe the three-dimensional structure of a crucial intermediate, which is probably a structural starting point (nucleus) in the fibril formation process. These results suggest that under our experimental conditions this crucial intermediate serves both as the fibrillation nucleus, as well as the elongating species. We propose that the latter intermediate is an interesting target for small molecules in order to prevent or reduce amyloid fibril formation.

## Abstract 3

Insulin fibrillation in solution is studied using small-molecule x-ray scattering. This study provides insights into the early stages of amyloid fibrillation, revealing the amyloid fibril repeat unit and evidence of a helical oligomer.

## Introduction

Amyloid fibrils are associated with critical diseases such as Alzheimer disease and Type 2 diabetes. In each amyloid disease, a particular protein or polypeptide aggregates and forms insoluble fibrils [1]. Moreover, amyloid fibrils play a critical role in unwanted degradation of a number of protein-based drugs [2,3]. Insulin fibrillation is a commonly used example of amyloid fibrillation because amyloid deposits have been observed in patients after subcutaneous insulin infusion [4] as well as in vitro, where insulin easily fibrillates, causing problems during production, storage, and delivery of insulin-based drugs [2].

The amyloid fibrils share several common structural properties, in which twisted and unbranched amyloid fibrils typically have a diameter of about 100 Å and highly variable lengths up to several microns [5–8]. Furthermore, mature fibrils are suggested to be composed of intertwined protofibrils built from two to three intertwining protofilaments, each with a typical diameter of 15–50 Å [6,7,9]. The most dominant repeating features of presumably all amyloid fibril structures are suggested cross-β-sheets with the β-strands running perpendicular to the fibril axis and an inter-strand spacing of about 4.8 Å [8,10]. Recently, atomic resolution structures of amyloid-like hexameric peptide segments, which form such cross-β-sheets, have been determined [11].

Amyloid fibrillation is proposed to be a nucleation-dependent process [12–16]. For insulin fibrillation, the initial step has been suggested to proceed through a non-native, partly unfolded, monomeric intermediate [12], which proceeds to form an oligomeric nucleus [17,18] prior to elongation of protofilaments. A more thorough knowledge of the structure and mechanism behind the formation of amyloid fibrils in general, and at the early stages of nucleus formation in particular, is essential for the understanding of the processes of amyloidosis. It is equally crucial for the rational design of drugs to inhibit or reverse amyloid formation, and for the general understanding of the mechanisms of protein folding and stability, since the ability to form amyloid fibrils has been proposed to be a generic feature of all polypeptide sequences [1]. Importantly, it has been suggested that oligomeric precursors of amyloid fibrils, and not the mature fibrils themselves, may be cytotoxic [19,20]. Indeed, there are indications that precursors are the main cause of toxicity [21]. This further emphasizes the importance of characterizing such oligomeric precursors, in the search for therapeutic treatment of amyloid diseases.

A structural characterization of the fibrillation nucleus is difficult, because at low protein concentrations, this nucleus is the thermodynamically least-favorable species [22]. However, a recent mathematical model of the nucleation-dependent fibrillation process suggests that the nucleus becomes thermodynamically stable and accumulates above the supercritical concentration [16]. The species is thus, per definition, no longer a thermodynamic nucleus, but is defined as a structural nucleus. The three-dimensional shape of the structural nucleus is thus identical to the thermodynamic nucleus present at low concentrations [16]. However, the structural nucleus is still inherently difficult to isolate and characterize. Most methods applied for biophysical or structural characterization of amyloid fibrillation are problematic because they either disturb the equilibrium of the fibrillation process (such as in x-ray crystallography or nuclear magnetic resonance), or the sample suffers from surface-mediated effects (such as in scanning tunneling microscopy, atomic force microscopy [AFM], and cryoelectron microscopy [cryo-EM]). In contrast, small-angle x-ray scattering (SAXS), which yields low-resolution macromolecular structures, allows examination of the fibrillation process directly in solution, so that the individual components present during the evolving equilibrium can be studied. We report a state-of-the-art SAXS analysis of fibrillating insulin and determine the ab initio low-resolution structure of the amyloid fibril repeating unit. Moreover, the structure of a helical oligomer, presumably representing the insulin structural nucleus, is determined, providing surprising details regarding its oligomeric composition and organization, with far-reaching conceptual impact on the current understanding of the early stages of amyloid fibrillation.

## Results

### Insulin above the Supercritical Concentration

**Figure 1:**
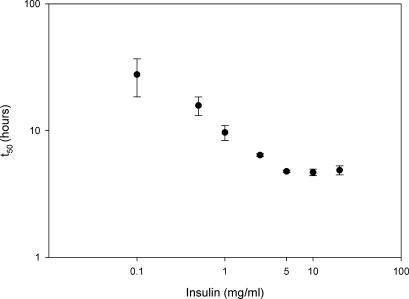
Estimation of the Supercritical Concentration The time required for a fibril formation reaction to reach 50% completion (*t*_50_) is plotted against total insulin concentration with both variables at logarithmic scales. The concentration used for SAXS analysis is 5 mg/ml. At 5 mg/ml, the rate of fibril formation is independent of the protein concentrations, hence, above the supercritical concentration.

Above a certain supercritical concentration, at which the rate of the fibril formation reaction becomes almost independent of the protein concentration, Powers and Powers demonstrate that the structural nucleus becomes more stable than the monomer [16]. The concentration dependency was measured using Thioflavin T (ThT) fluorescence. The experimental conditions used in our SAXS analysis, with insulin concentrations of 5 mg/ml, are above the supercritical concentration (Figure 1).

### X-ray Diffraction Pattern of Insulin Fibrils

A characteristic x-ray fiber diffraction pattern was observed for insulin samples under the experimental conditions employed during the SAXS studies (Figure S3). A typical meridional signal is seen at 4.8 Å, and an equally typical equatorial signal is recorded at 10.5 Å. In addition to the characteristic ThT fluorescence signal, this confirms the existence of fibrils.

### A Distinct Oligomer Elongates the Maturing Fibrils

**Figure 2:**
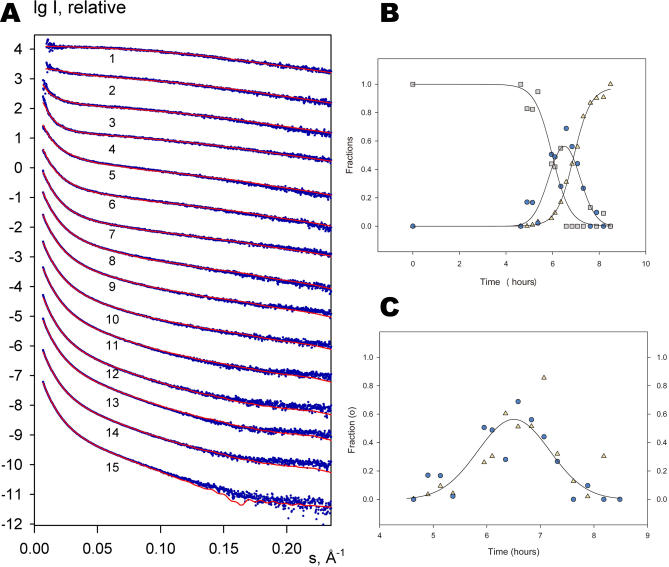
The Insulin Fibrillation Process (A) SAXS patterns from the insulin solution as a function of time (the successive curves labeled 1 to 15 are displaced one logarithmic unit down for clarity). Top curve, monomeric insulin, and bottom curve, mature fibril. The time points correspond to the time points visible in (B) and (C). Blue dots represent experimental data, and red lines show fits from the three-component mixtures (for the bottom curve, fit by the ab initio shape model is displayed). (B) Relative volume fractions of the three components present during the insulin fibrillation process. The insulin monomer curve is shown as grey squares, the helical oligomer curve as blue circles, and the fibril curve as beige triangles. (C) Comparison of the fraction of oligomer (blue circles and fitted curve) and the rate of fibril formation evaluated by the first derivative of the fibril fraction curve (beige triangles) as a function of time.

Time-resolved synchrotron SAXS was applied to study the insulin fibrillation process in solution, starting from monomeric insulin. During the 5-h lag phase, only monomers were detected (Figure 2 and Table S1), with a small increase in the average size of the molecules towards the end of the lag phase, presumably reflecting the expected partial unfolding of a fraction of the monomers. During the 4-h elongation phase, a significant growth of particles is observed leading to drastic changes in the scattering pattern (Figure 2A). Importantly, the scattering curves recorded during the elongation phase could not be described by linear combinations of the scattering from monomers and mature fibrils. Singular value decomposition (SVD) analysis further confirmed that three components contributed significantly to the scattering during the elongation phase (Figure S1). This suggested to us the presence of monomers, fibrils, and a distinct oligomer, apparently representing the hitherto largely uncharacterized structural nucleus. The scattering pattern from the distinct oligomer (Figure S2A) was evaluated as described in Materials and Methods. All the individual scattering patterns collected during fibrillation could be fitted by a linear combination of the three components yielding their volume fractions (Figure 2A and 2B). As expected, the amount of monomeric insulin decreases gradually. Appearance of large amounts of the distinct oligomer accompanies the monomer decrease. There is an additional lag phase of about 1 h between the initial observation of oligomer formation and the onset of fibril growth. Most remarkably, the growth rate of the mature fibrils is proportional to the amount of the oligomer in solution (Figure 2C), suggesting that the oligomers represent the building blocks for the growing fibrils. Thus, above the supercritical concentration, the insulin fibril elongation likely proceeds primarily by addition of oligomers. Also, the amount of monomers in solution is close to zero during the later stages of fibril formation, which further supports the notion that oligomer addition elongates the protofilaments. This mechanism is further supported by the shape of these oligomers, presented below.

### The Distinct Oligomer Is a Helical Bead-on-a-String Assembly

**Figure 3:**
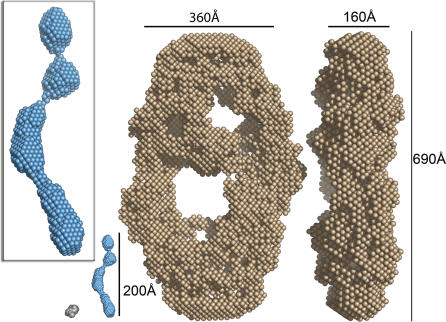
The Three Components Present during the Insulin Fibril Elongation Phase at Relative Scale The insulin monomer is shown in grey in the lower left corner, the ab initio structure of the helical oligomer in blue, and the ab initio structure of the repeating helical unit of the fibril in beige (in front and side view). The inset shows the oligomer in a zoom box. Three intertwining protofibrils appear from the ab initio model of the repeating unit of the fibril. Overall dimensions are marked on the figure. Individual spheres in the oligomer and the repeating helical unit represent the smallest units during ab initio modeling.

As early as in 1957, Waugh proposed that a nearly simultaneous interaction of three to four insulin monomers forms a nucleus [18], and later mass spectroscopy also revealed a relatively small nucleus [17]. An elongated association of monomers prior to fibrillation has been observed by AFM on insulin samples [23]. The existence of significant amounts of an oligomeric species of insulin prior to the appearance of mature fibrils and throughout the fibril elongation process was also detected using dynamic light scattering [24] and time-lapse AFM [25]. In the present study, we further characterize the low-resolution shape of the oligomer ab initio that yields a good fit to its scattering pattern (Figure S2A) and reveals a helical structure with a length of 200 Å and a twist of 270°, or three quarters of a full helical turn in the direction of the long axis (Figure 3). The hydrated volume of the oligomer is 6.4 × 10^4^ Å^3^, corresponding to an approximate molecular weight of 32 kDa, or 5.6 insulin monomers. Visually, the helical oligomer also appears as a bead-on-a-string assembly of six units, each with dimensions comparable to those of insulin monomers (Figure 3). Moreover, using independent rigid-body modeling (Protocol S1) in terms of the crystallographic model of partially unfolded insulin at pH 2 [26], the models consisting of five or six subunits provide the best fit to the scattering pattern from the distinct oligomer (Figure S2A). A typical rigid-body model (Figure S2B) displays six monomers in a helix-like configuration, very similar to that observed in the ab initio model (Figure 3).

### The SAXS Solution Structure of the Repeating Unit of Insulin Mature Fibrils

**Figure 4:**
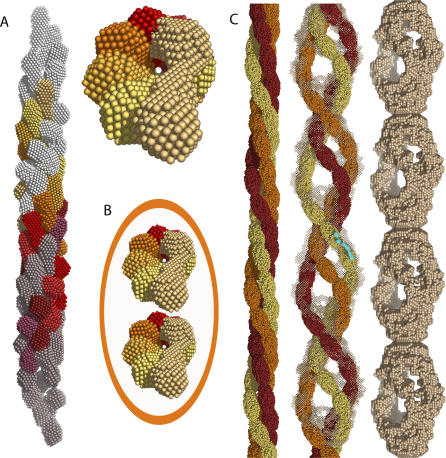
Model for Assembly of the Distinct Insulin Oligomers into Protofilaments, Protofibrils, and Fibrils (A) Eight helical oligomers (shown in a color scale from purple through red to light yellow) intertwine by simultaneous rotation and translation to form the protofilament. Eight additional precursors in grey nuances form the open ends of the model. Side and top views are shown. (B) Two protofilaments presumably intertwine to form the protofibrils, with an average diameter of 100 Å (top view, intertwining not depicted). An orange ellipse marks the outer boundaries of one protofibril. (C) Three protofibrils (shown in orange, yellow, and red) intertwine to form the mature insulin amyloid fibril. Shown are side and front views. Four repeats of the ab initio SAXS structure of the coherently scattering unit of the fibril (Figure 3) are shown in beige semi-transparent dots superimposed on the front view as well as separately in solid spheres. The latter is rotated 25° around the elongating axis compared to the front view. An oligomer (blue) is superimposed onto the fibril model. See Videos S1 and S2 for two movies visualizing the model of protofilament and fibril assembly. The figures were generated using PyMol (DeLano Scientific).

The SAXS data collected after 9-h incubation were used to evaluate the shape and dimensions of the mature fibril. Given the resolution of the scattering pattern (from about 900 Å to 12 Å), the SAXS data provide information about the organization of the coherently scattering volume inside the fibril, associated with its repeating unit. Using indirect Fourier transformation (IFT), the repeating unit has a length of about 700 Å and a cross-section diameter of about 300 Å (Tables S1 and S2). Ab initio modeling of the fibril was further performed based on these dimensions, showing an elliptically shaped repeating unit with overall dimensions of 690 × 390 × 160 Å^3^ (for a fit of the final model and data, see the lowest curve in Figure 2A). The length (690 Å) of this unit is within the range of helical crossovers of 350 to 940 Å observed with cryo-EM of insulin fibrils [6]. The fibril consists of three intertwining helical protofibrils (Figures 3 and 4), as also observed by cryo-EM and AFM [6,7]. Each protofibril has an average diameter of about 100 Å, a protofibril diameter that is in accordance with cryo-EM studies [6]. The fibril has a left-handed helical twist as observed for most amyloid fibrils [6,27,28]. The hydrated volume of the repeating helical unit of the fibril (1.44 × 10^7^ Å^3^), corresponds to an approximate molecular weight of 7,200 kDa or about 1,240 insulin monomers.

Given the potential heterogeneity of the mature fibrils and possible residual contribution of the monomers and oligomers even at the latest stages of fibrillation, the models (Figures 3 and 4) represent average shapes of the most abundant fibrillating units. It should be noted, however, that the heterogeneity of the fibrils appears to be low, given that there are only three components in the above model-free SVD analysis. Note also that the potential heterogeneity of the mature fibrils does not challenge our results concerning the presence and volume fractions of the oligomeric species, since these have been determined independently of the model of mature fibrils.

### A Mechanism for Elongation of Insulin Fibrils

Although one cannot completely exclude the possibility that fibrils may grow by addition of individual insulin monomers, the fact that the growth rate is proportional to the amount of oligomers strongly suggests that incorporation of the latter is the predominant mechanism of elongation. Moreover, the helical shape of the oligomer (Figure 3) is ideally suited for entering a protofilament. We have generated a tentative model of the arrangement of intertwining helical oligomers into dense protofilaments, assuming that elongation of protofilaments is directed by oligomer association. Figure 4 displays such a model with a central cavity in which the cross-section contains eight helical oligomers (see Video S1 for the stepwise construction of the protofilament by the addition of helical oligomers). Fourteen repeats of the length of these eight helical oligomers would form a full turn of one protofilament across three fibril repeating units. Two protofilaments presumably intertwine to form the protofibrils, yielding an average protofibril diameter of about 100 Å. Finally, three protofibrils intertwine to form the mature fibril (see Video S2 for a movie on the stepwise construction of the mature fibril from protofilaments). Hence, a total of 224 oligomers would build up one full turn of the protofibril. When following one single protofibril across three repeating fibril units, the protofibril will have traversed all three positions of the three strands that are visible within one fibril repeat. Thus, the volume and estimated molecular weight of the coherently scattering unit must correspond to the calculated number of 224 helical oligomers that define our structural model, which indeed is the case, and the suggested packing is in agreement with the molecular weights of 32 and 7,200 kDa of the oligomer and the helical repeat of the mature fibril, respectively. In the suggested packing of the protofilament, the helical oligomer is preshaped for interactions with other oligomers, and all subsequent oligomer additions elongate the protofilament. Moreover, oligomer additions are possible in both directions. Thus, our model suggests that above the supercritical concentration, the helical oligomer is both the structural nucleus and the elongating building block of insulin amyloid fibrils.

## Discussion

The existence of oligomeric species during the amyloid fibrillation process is not unique for insulin fibrillation and has previously been reported, e.g., for β-amyloid peptide [29], β-2-microglobulin [30], phosphoglycerate kinase [31,32], a fragment of Syrian hamster prion protein [32,33], and myoglobin with polyglutamine insertions [34]. The overall sizes of some of these aggregates are compatible with the insulin oligomer described in this study. An insulin fibrillation study using dynamic light scattering also reports significant amounts of oligomers of similar size [24]. Interestingly, the β-amyloid peptide oligomers revealed by scanning tunneling microscopy appeared as a bead-on-a-string–like assembly of individual monomers [29], strikingly similar to the present model of insulin oligomers. Thus, the new structural findings presented here may reveal generic features of amyloid fibrillation.

The importance of monomeric insulin for fibrillation has been reported in many studies [12,13] and explained by the need for a partial unfolding of the monomer prior to association into amyloid fibrils. This is corroborated by Fourier transform infrared spectroscopy and circular dichroism studies, showing that a significant conversion of α-helical to β-sheet structure accompanies formation of insulin amyloid fibrils [2,12]. The presence of repeating cross-β-sheets perpendicular to the fibril axis with an inter-strand spacing of about 4.8 Å in the present insulin fibrils was confirmed by x-ray diffraction (Figure S3). In the case of the oligomers of β-amyloid peptide [29], which have a similar appearance to the insulin structural nucleus, β-strands are directly observed [29], and a structural conversion accompanying oligomer formation is also observed for insulin [24]. However, it remains to be shown whether the structural conversion of insulin to a β-structure occurs prior to or during structural nucleus formation, because insulin monomers substantially refolded into β-structure have never been isolated. Under amyloidogenic conditions, a partly modified structure has been described, although a central natively folded core remains [35].

Assuming that a β-sheet with strands separated by 4.8 Å runs parallel to the long axis of the distinct helical oligomer with a length of 200 Å, this corresponds to approximately 40 β-strands with a relative twist of each β-strand in the β-sheet of about 7°. Based on this rationale, each monomer of the oligomer should accommodate six to seven β-strands. Small peptide segments of only three or four residues and several hexa-peptides from insulin have been shown to form amyloid fibrils [36] and crystals with amyloid features [37], respectively. This suggests that insulin is, at least in theory, able to adopt such a structure, as it has 51 amino acids. It is known that the two interchain and one intrachain disulfide bonds in insulin are accommodated in fibrils [6], and it remains to be shown how insulin is able to accommodate this in such a β-sheet–rich structure. Another issue to consider is the recent theoretically based suggestion that α-sheets, and not β-sheets, may form the repeating backbone in the oligomer [38,39]. These conformations cannot be distinguished within the helical insulin oligomer due to the limited resolution of the SAXS data.

The nucleation polymerization model suggests that partially unfolded monomers form an oligomeric nucleus, followed by the elongation process proceeding via direct addition of structurally non-native monomers [12,40]. However, in our studies, performed above the supercritical concentration, the oligomeric species is present in high amounts during fibrillation, and the amount is proportional to the rate of fibril elongation. Although Powers and Powers [16] consider elongation only via monomer addition, they also note that it seems physically realistic that the oligomers would associate. This is further supported by the observation of β-sheets in fibrillar oligomers [29]. Although elongation of protofilaments by helical oligomer association appears structurally feasible, we cannot strictly rule out that elongation occurs at least in part through monomer addition. This would, however, imply that the oligomer dissociates into monomers at rates comparable to the elongation rate of the growing fibrils. Thus, the amount of oligomers would decrease prior to or simultaneous with the appearance of fibrils [16]. In contrast, we observe the highest amount of oligomers not at the start of the fibril growth, but rather when the rate of elongation is maximal. The elongation of fibrils also continues after the monomer concentration is no longer detectable, serving as further argument in favor of the “elongation via the structural nucleus” mechanism.

An elongation through oligomer assembly was proposed for a peptide derived from the prion protein Sup35 [41]. Fluid oligomeric species was observed and hypothesized to be structurally distinct from the nucleus. The oligomeric species was suggested to change conformation upon interaction with the nucleus, thus speeding up the assembly process. It is unclear whether the experiments by Serio et al. [41] were performed below supercritical concentration or, like ours, above the supercritical concentration, because the supercritical concentration, to our knowledge, has not yet been established for Sup35. The oligomeric species we observe does accumulate above the supercritical concentration (and thus according to Powers and Powers [16] should correspond to the structural nucleus), and it reveals a distinct helical shape strikingly suitable to associate with other helical oligomers (see Video S1). Of course a conformational change may happen during the association of the structural nuclei in the elongating protofilaments, but our modeling shows that such a conversion is not required for the elongation of protofilaments via the assembly of structural nuclei.

In summary, we present experimental evidence for the accumulation of a helical oligomeric species, which has previously been suggested to be the structural nucleus. In contrast to the numerical simulation of elongation via monomer addition proposed by Powers and Powers, our experimental results demonstrate that the amount of the structural nucleus in solution determines the rate of elongation. The shape of the structural nucleus is uniquely well suited for forming the protofilaments, obviating the need for conformational changes in the nucleus before associating into the growing protofilaments.

A secondary nucleation mechanism after the primary nucleus formation has been proposed for insulin fibrillation [23,42,43]. Such mechanisms may be branching, fragmentation, and nucleation on the fibril surface. These features would not be distinguishable in the current study, since the scattering units present in solution at all times would still be oligomers and mature fibril repeating units, irrespective of the site of secondary nucleation.

Our model of a dense protofilament structure has a central cavity (Figure 4A and 4B). Such a cavity has been proposed in some, but not all, models of amyloid protofilaments [44–46], e.g., in a β-helical model for poly-glutamine [47] and in hollow nanotube structures observed for amyloid fibrils, e.g., Aβ(1–40) [48] and Aβ(1–25) [49]. Although no high-resolution structure of a mature amyloid fibril is currently available, it appears that amyloid fibrils possess common structural features, the most dominant being the high content of β-sheets with β-strands perpendicular to the longest and elongating axis of the fibril [8]. This might suggest a common structural principle for the mechanism of fibril formation for several amyloid proteins. The helical oligomer preshaped for fibril formation could represent a generic principle for the building blocks in amyloid fibril formation, which also may represent the structural scaffold followed during monomer addition at subcritical concentrations.

This study demonstrates the power of SAXS analysis on data from amyloid systems, since it is possible to separate scattering contributions from different species present during fibrillation. The method allows determination of both the amount and the low-resolution structure of the species in solution. For amyloid systems characterized by nucleated polymerization, the thermodynamically unfavorable nucleus becomes stable above supercritical concentrations, and is defined as a structural nucleus [16]. Here, we report experimental evidence for the accumulation of the structural nucleus in insulin fibrillation and characterize its structure. This oligomer has a distinct helical structure in solution, and the correlation between the amount of helical oligomer present in solution and the growth rate of the mature fibrils suggests that it is the oligomer that primarily elongates the growing protofilaments. Using supercritical concentrations, the potential toxicity of accumulating oligomers of various amyloid proteins could be investigated. The oligomer is conceptually central for the understanding of the early stages of amyloid fibrillation. The low-resolution model and documented presence of large amounts of oligomers can facilitate the search of new strategies for preventing or reversing insulin fibrillation or amyloid fibrillation in general, e.g., by hindrance of structural nucleus formation and/or packing of the helical oligomer into protofilaments.

## Materials and Methods

### Materials.

Human zinc insulin was obtained from Novo Nordisk, http://www.novonordisk.com. All other chemicals were of analytical grade.

### Determination of supercritical concentration.

A total of 0.06–12-mg/ml insulin in 20% acetic acid (pH 2.0), with 0.5 M sodium chloride was fibrillated at 45 °C on a Fluostar Optima plate reader from BMG Labtechnologies (http://www.bmg-labtechnologies.com).

### X-ray diffraction.

Insulin (5 mg/ml) in 20% acetic acid (pH 2.0), with 0.5 M sodium chloride was fibrillated at 45 °C on a Fluostar Optima plate reader from BMG Labtechnologies. Fibrils were centrifuged at 18,000 *g* on a Hettich centrifuge and dried in a desiccator. X-ray synchrotron data were collected at MAXLAB beamline 911–3, Lund, Sweden, using a MarMosaic 225 detector at 20 °C; λ = 1.3 Å, and a 350-mm specimen:detector distance during 30-s exposure time.

### SAXS data collection and reduction.

A pellet of human insulin was dissolved at 5 mg/ml in 20% acetic acid with 0.5 M sodium chloride, at *t* = 0 (Table S1). The sample was kept and measured at 45 °C approximately every 15 min. Zinc acetate was added to the background buffers corresponding to two Zn^2+^ ions per insulin hexamer in the protein samples. Synchrotron scattering data were collected on the X33 camera of the European Molecular Biology Laboratory on DORIS III (Deutsches Electronen Synchrotron [DESY], Hamburg, Germany) at a wavelength of 1.5 Å, using a MAR345 Image Plate detector, in the momentum transfer range 0.01 < *s* < 0.50 Å^−1^ (*s* = 4π sinθ/λ, where θ is half the scattering angle) with 3-min exposure times. Selected insulin samples exposed repetitively did not result in detectable radiation damage. Data analysis was performed using the software suite ATSAS 2.1 [50]. The data reduction was done using the program PRIMUS [51], and the molecular masses of the solutes were estimated by comparing the extrapolated forward scattering *I*(0) with that of a reference solution of bovine serum albumin (Table S1). IFT were evaluated by the program GNOM [52], providing estimates of distance distribution functions, including the radii of gyration *R*_g_ and maximal distances (*D*_max_) within the particles.

### SVD analysis and decomposition of data curves into monomers, oligomers, and fibrils.

SVD analysis was performed by the program SVDPLOT [51] using all the measured datasets to detect the number of independent components (Figure S1). The program OLIGOMER [51] was employed to represent the experimental data by linear combinations of the scattering from individual components (monomers, oligomers, and fibrils) and to compute their volume fractions (Figure 2B). The calculated scattering from a monomer of insulin (partially unfolded monomers at acidic pH, pdb-file 1GUJ) and the low-pass–filtered experimental scattering from the coherent unit of the fibril after 9-h incubation were employed. To find the first representation of the scattering pattern from the oligomer, several tentative models were built assuming the oligomer to be from dimers to hexadecamers constructed in long, flat, zigzag, or compact compositions. The representation was selected for which OLIGOMER yielded overall best agreement between the experimental data measured during the elongation phase and the linear combinations of the three components. Then the volume fraction of the oligomer was set to zero, and those of the monomers and fibrils were kept at the values for the three-component fit. The scattering from the oligomer was computed as the average of all the residuals between the experimental data and two-component fits. This decomposition ensures that the scattering curve representing the oligomer is independent of potential heterogeneity of the mature fibrils, because the contribution of the latter is removed. Thus, the determination of the volume fractions during fibrillation and the modeling of the structural nucleus are independent both of the heterogeneity of the mature fibrils and of the modeling of the helical fibril repeat.

### Modeling of insulin fibril and oligomer.

Ab initio shapes of the insulin fibril repeat and the distinct oligomer were obtained using the program DAMMIN [53]. The program employs a simulated annealing protocol to search for a compact bead model minimizing the discrepancy between the experimental and calculated curves at low resolution (up to *s* about 0.1–0.2 Å^−1^). Based on the best approximating three-parameter body as computed by the program BODIES [51], the search volume for the fibril repeat was an ellipsoid with half-axes of 350, 250, and 150 Å using 35,000 spheres. Individual jobs were loaded into a Linux cluster, and 20 independent models were averaged using the program DAMAVER [54]. The resulting model was used as input for subsequent runs of 20 individual models, for a total of five refinements. Likewise, the oligomer shape was calculated inside a sphere with a diameter of 200 Å, and gradually refined over three runs using an average of 20 individual models as input. The final averaged and filtered models also yielded the excluded volume of the hydrated particle.

## Supporting Information

Figure S1 Singular Value Decomposition (A) A plot of eigenvalues as a function of the ordinal number of the component; the number of significant eigenvalues corresponds to the number of species significantly contributing to the scattering data.
(B) A plot of the first six eigenvectors normalized by the appropriate eigenvalue. Successive eigenvectors are displaced by unity along the vertical axis for clarity, with the first eigenvector displayed at the top and the last at the bottom.
It is seen in (A) that the first three eigenvalues significantly exceed the rest, and in (B), that the eigenvectors starting from the fourth from the top contain essentially noise components. Both plots thus show that three scattering species are present in the solutions.
(29 KB PDF)

Figure S2 Fit of the Models of the Insulin Oligomer to the Scattering Data (A) Scattering from the oligomer (blue) and the computed curves from its ab initio model (red curve; model shown in Figure 3) and from the typical rigid-body model containing six insulins (green curve; model shown in surface representation in [B]). Partial overlap between monomers 5 and 6 may be caused by the use of only partly unfolded monomers, which are still mainly α-helical. Individual monomers in the oligomer are expected to consist of mainly β-structure and thus be significantly different than the native-like monomers used in this analysis. However, no radically unfolded monomer structures have been described, for which reason, native-like monomers are used. The resulting composition still has a striking resemblance to the ab initio low-resolution model, thus confirming the validity of the ab initio model.
(54 KB PDF)

Figure S3 X-ray Diffraction of Insulin Amyloid Fibrils X-ray diffraction pattern of dry insulin amyloid fibrils prepared in 20% acetic acid (pH 2.0) and 0.5 M NaCl. The diffraction pattern shows a dominant reflection at 4.8 Å on the meridian (M) and reflections at 10.5, 14.5, and 30 Å on the equator (E).
(218 KB TIF)

Protocol S1 SASREF Rigid-body modeling was used as a complementary approach to oligomer ab initio modeling, and the program SASREF was employed. SASREF [55] uses simulated annealing to optimize the configuration of subunits while fitting to the SAXS data. Four to seven insulin monomers were tested, even though native monomers are *not* expected to form the oligomer, and five or six monomers gave the best fit. Most importantly, the helical organization of subunits was recurring, thus confirming the ab initio DAMMIN shape.
(21 KB DOC)

Table S1 Overall Physical Parameters from Individual SAXS Data Files *R*_g_ and *I*(0) have been calculated using GNOM also providing estimates of maximal distances (*D*_max_) within the scattering particles. Molecular masses are estimated by comparing extrapolated forward scattering *I*(0) with that of a reference solution of bovine serum albumin. *R*_g_ was also calculated from Guinier approximation. The data from 308 min and on do not have the correct Guinier range, i.e., estimates are approximate.
(45 KB DOC)

Table S2 Optimizing the IFT For an optimal IFT, a systematic variation of *D*_max_ was made. The table shows the overall estimate of the distance distribution functions, as calculated in GNOM. A *D*_max_ of 70 nm was chosen and used for the ab initio modeling in accordance with the approximation from BODIES. However, for completeness, in the subsequent modeling, the elliptical initial search volume in DAMMIN was varied between 35–45 nm, 20–30 nm, and 15–25 nm for the half-axes *a, b,* and *c,* respectively, yielding best fits using half-axes of 350, 250, and 150 Å.
(36 KB DOC)

Video S1 Build-Up of Protofilament from the Helical Oligomer The assembly of the helical insulin oligomer (structural nucleus) into a protofilament is shown. Sixteen helical oligomers enter the growing protofilament one by one, and thereby elongate the growing protofilament. It is evident that the oligomer is preshaped for protofilament formation. Individual oligomers are colored on a color scale from dark violet through red, orange, and yellow to white. The eight central oligomers are fully packed against other oligomers, whereas each end of the protofilament has four oligomers that are open to form contacts with further elongating oligomers. For simplicity, only single oligomers are shown entering the protofilament ends. Note that the colloidal coagulation theory, as described by Smoluchowski [56], is compatible with the model. The shape of the oligomer makes it possible that each individual oligomer can associate with either single oligomers or multimers of oligomers, just as multimers of oligomers can associate end to end.
(3.2 MB MPG)

Video S2 Formation of Fibrils from Protofilaments The protofilament is rotated 360° and, subsequently, 90° to show the top view. A second protofilament is shown in the same view. The two protofilaments presumably intertwine to form the protofibril. The view changes after zooming in to show the protofibril surface in orange. The view rotates back 90° and then zooms out. Then the three intertwining protofibrils are shown in orange, red, and yellow, respectively, during two 360° rotations. Following that, four ab initio models of the fibril helical repeat (the coherently scattering unit) are superimposed in grey during four full rotations, changing the view to include or exclude the model of the three intertwining protofibrils. The movie was generated using PyMol (DeLano Scientific, http://www.pymol.org).
(2.0 MB MPG)

## Abbreviations

Aβ: amyloid beta
cryo-EM: cryo-electron microscopy
*D*_max_: maximal distance (of the pairwise distances probability distribution)
IFT: indirect Fourier transformation
*R*_*g*_: radius of gyration
SAXS: small-angle x-ray scattering
SVD: singular value decomposition
