# Supplementary material for: A Helical Structural Nucleus Is the Primary Elongating Unit of Insulin Amyloid Fibrils
Source: PLoS Biol. 2007 May 1;5(5):e134. doi: 10.1371/journal.pbio.0050134 (PMC1858711; doi:10.1371/journal.pbio.0050134)
Supplement: Supplementary file 2 [file pbio.0050134.sg002.pdf]

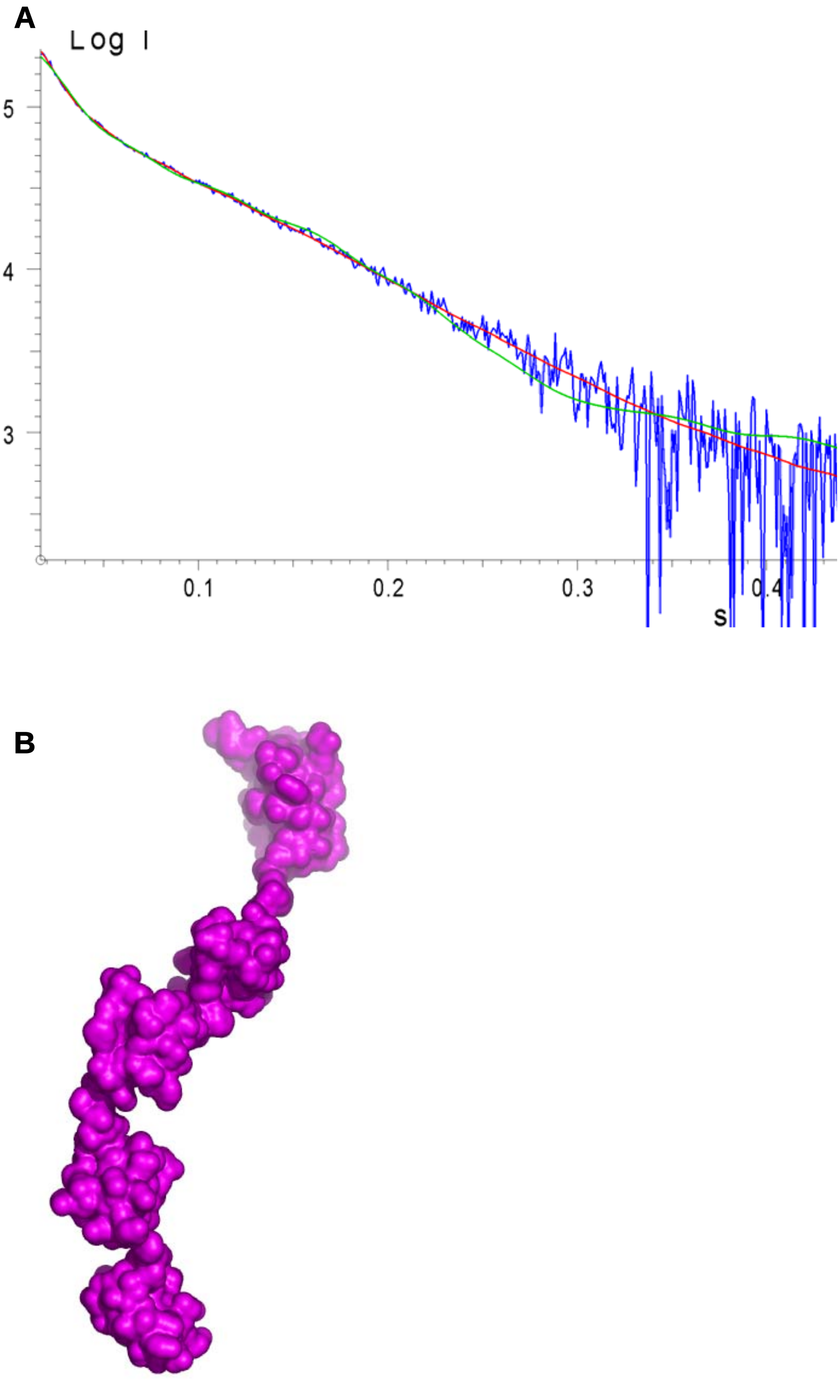
A
Log I
5
4
3
0.1
0.2
0.3
0.4
s
B
