# Supplementary material for: A Helical Structural Nucleus Is the Primary Elongating Unit of Insulin Amyloid Fibrils
Source: PLoS Biol. 2007 May 1;5(5):e134. doi: 10.1371/journal.pbio.0050134 (PMC1858711; doi:10.1371/journal.pbio.0050134)
Supplement: Supplementary file 3 [file pbio.0050134.sd001.doc]

**Supporting Materials and Methods**

**Sasref.** The rigid body modeling was used as a complementary approach to oligomer *ab initio* modeling and the program SASREF [1] was employed. SASREF uses simulated annealing to optimize the configuration of subunits while fitting to the SAXS data. Four to seven insulin monomers were tested, even though native monomers are *not* expected to form the oligomer, and five or six monomers gave the best fit. Most importantly, the helical organization of subunits was recurring, thus confirming the *ab initio* DAMMIN shape.

**Protocol S1, Reference**

Reference List

1.	Petoukhov MV, Svergun DI (2005) Global rigid body modeling of macromolecular complexes against small-angle scattering data. Biophys J 89: 1237-1250.
