# Supplementary material for: A Helical Structural Nucleus Is the Primary Elongating Unit of Insulin Amyloid Fibrils
Source: PLoS Biol. 2007 May 1;5(5):e134. doi: 10.1371/journal.pbio.0050134 (PMC1858711; doi:10.1371/journal.pbio.0050134)
Supplement: Supplementary file 4 [file pbio.0050134.st001.doc]

**Table S1**

| Minutes | R_g_ (nm) | I(0) (x1000) | Average Molecular Weight (kDa) |
| --- | --- | --- | --- |
| 0 | 1.09 | 123 | 5.9 |
| 63 | 1.07 | 128 | 6.1 |
| 92 | 1.12 | 120 | 5.8 |
| 154 | 1.13 | 118 | 5.7 |
| 180 | 1.15 | 129 | 6.1 |
| 217 | 1.15 | 131 | 6.3 |
| 245 | 1.14 | 128 | 6.6 |
| 294 | 2.23 | 182 | 8.7 |
| 308 | 8.74 | 495 | 23.7 |
| 322 | 20.5 | 4220 | 202 |
| 352 | 15.6 | 3460 | 166 |
| 366 | 18.1 | 6250 | 300 |
| 381 | 21.1 | 14500 | 695 |
| 396 | 22.3 | 32000 | 1530 |
| 410 | 22.6 | 56800 | 2720 |
| 424 | 22.9 | 71800 | 3440 |
| 440 | 23.6 | 105000 | 5040 |
| 457 | 23.9 | 123000 | 5900 |
| 474 | 23.5 | 193000 | 9260 |
| 492 | 23.4 | 168000 | 8060 |
| 509 | 23.4 | 144000 | 6900 |
