# Supplementary material for: A Helical Structural Nucleus Is the Primary Elongating Unit of Insulin Amyloid Fibrils
Source: PLoS Biol. 2007 May 1;5(5):e134. doi: 10.1371/journal.pbio.0050134 (PMC1858711; doi:10.1371/journal.pbio.0050134)
Supplement: Supplementary file 5 [file pbio.0050134.st002.doc]

**Table S2**

| D_max_ (nm) | Overall Estimate |
| --- | --- |
| 50 | 0.66 |
| 55 | 0.66 |
| 60 | 0.85 |
| 65 | 0.88 |
| 70 | 0.96 |
| 75 | 0.91 |
| 80 | 0.87 |
| 85 | 0.75 |
| 90 | 0.59 |
| 95 | 0.57 |
| 100 | 0.56 |
| 105 | 0.57 |
| 110 | 0.55 |
| 115 | 0.60 |
| 120 | 0.48 |
| 125 | 0.61 |
| 130 | 0.65 |
| 135 | 0.32 |
| 140 | 0.32 |
| 145 | 0.32 |
